# Epidemiologically characteristics of human brucellosis and antimicrobial susceptibility pattern of *Brucella melitensis* in Hinggan League of the Inner Mongolia Autonomous Region, China

**DOI:** 10.1186/s40249-020-00697-0

**Published:** 2020-06-29

**Authors:** Hai-Tao Yuan, Cheng-Ling Wang, Li-Na Liu, Dan Wang, Dan Li, Zhen-Jun Li, Zhi-Guo Liu

**Affiliations:** 1 Hinggan League Center for Disease Control and Prevention, Ulanhot, 137400 China; 2 State Key Laboratory for Infectious Disease Prevention and Control, National Institute for Communicable Disease Control and Prevention, Chinese Center for Disease Control and Prevention, Beijing, 102206 China; 3 Hinggan League people’ hospital, Ulanhot, 137400 China

**Keywords:** Brucellosis, Epidemiology characteristic, *Brucella melitensis*, Genotyping, Antimicrobial susceptibility, Hinggan league, Inner Mongolia Autonomous Region

## Abstract

**Background:**

Hinggan League is located in the Northeast of the Inner Mongolia Autonomous Region, the historically endemic area of animal and human brucellosis. In this study, the epidemiological characteristics of human brucellosis were analyzed, and the genotypic profile and antimicrobial susceptibilities of *Brucella melitensis* strains isolated from humans in Hinggan League were investigated.

**Methods:**

The epidemic characteristics were described using case number, constituent ratio, and rate. The 418 human blood samples were collected and tested by bacteriology, and suspect colonies were isolated and identified by conventional biotyping assays, the VITEK 2.0 microbial identification system, and AMOS (*Brucella abortus*, *B. melitensis*, *B. ovis*, and *B. suis*)-PCR. Subsequently, all strains were genotyped using multiple-locus variable-number tandem repeat analysis (MLVA) assays, and the antimicrobial susceptibility pattern of *Brucella* strains against the 10 most commonly used antibiotics was determined by microdilution method.

**Results:**

A total of 22 848 cases of human brucellosis were reported from 2004 to 2019, with an annual average incidence of 87.2/100 000. The incidence rates in developed areas of animal husbandry (Horqin Youyi Qianqi [161.2/100 000] and Horqin Youyi Zhongqi [112.1/100 000]) were significantly higher than those in forest areas (Arxan [19.2/100 000]) (*χ*^2^ = 32.561, *P* < 0.001). In addition, peak morbidity occurred during May–August, accounting for 72.6% (16582/22 848) of cases. The highest number of cases occurred in the 40+ age group, accounting for 44.4% (10 137/22484) of cases, and morbidity in males was significantly higher than that in females in all age groups (χ^2^ = 299.97, *P* < 0.001), the most common occupation was farmers. A total of 54 *B. melitensis* strains were divided into 37 genotypes (GT1–37) with 80–100% genetic similarity. All 25 strains were sensitive to seven tested antibiotics, phenotypic resistance to cotrimoxazole and azithromycin was observed in 5 (20%) and 25 (100%) of the isolates, respectively.

**Conclusions:**

Human brucellosis exhibited a significant increasing trend and *B. melitensis* is the main pathogen responsible for human brucellosis in this region. Improved surveillance of infected animals (sheep) and limiting their transfer and trade are optional strategies for decreasing the incidence of this disease.

## Background

Brucellosis, caused by bacteria of the genus *Brucella,* is a prevalent zoonotic disease with a high socioeconomic and economic burden [1]. The disease can be transmitted from animal reservoirs, such as cattle, sheep and pigs, to humans primarily through direct contact with infected animals and consumption of contaminated food. The symptoms of infection include abortion, infertility, decreased production, and lameness in animals [2]. However, in humans, the disease can manifest as undulating fever with arthralgia, sometimes associated with chronic and severe complications, such as orchitis, spondylitis, and arthritis [3, 4]. People, especially those who engage in husbandry activities, slaughtering and livestock trading, are at higher risk of developing brucellosis.

A high morbidity level of brucellosis in humans and animals has been found in some regions of China [5]. Although brucellosis is mainly an animal disease, globally, more than 500 000 human cases are reported each year [6]. Hinggan League is located in the Northeast of Inner Mongolia Autonomous Region and is an important husbandry base, with a population of 1.604 million; it has jurisdiction over three banners, one county, and two cities. Hinggan League was an old endemic area of animals and human brucellosis, and brucellosis was distributed in all six regions (counties and cities). New brucellosis infections in humans in this region have increased dramatically in the last decade [7]. From an epidemiological perspective, *B. melitensis* strains are the predominant species in the Hinggan League. The spread of the brucellosis epidemic not only results in heavy losses for the production of husbandry but also poses a threat to human health and safety [8, 9]. Multiple-locus variable-number tandem repeat analysis (MLVA) is widely used in epidemiological investigations of *Brucella* infections, as this analysis effectively discriminates strains and generates results that are largely in agreement with the genotypes identified using whole genome sequence (WGS)-single nucleotide polymorphism (SNP) analysis [10]. However, WGS-SNP analysis is a more powerful and reliable method of discerning *Brucella* strains [11]. Brucellosis treatments are often empirical, because little is known about the antibiotic susceptibility of *Brucella* spp. in this region. To date, a comprehensive test of the antimicrobial susceptibility of human *B. melitensis* isolates from Inner Mongolia and China has not been performed. Previous studies have shown that 31 *B. melitensis* isolates from Liaoning Province were resistant to azithromycin and clarithromycin [12], and a few strains from Ulanqab, Inner Mongolia were resistant to rifampin and cotrimoxazole [13]. Liaoning Province, Ulanqab, and Hinggan League are geographically close. Consequently, this study investigated the 22 848 human cases reported in Hinggan League of Inner Mongolia Autonomous Region in the period of 2004–2019. Genotyping and antimicrobial susceptibility testing were performed in *Brucella* strains isolated from 2018 to 2019 to better understand the distribution characteristics and epidemic trend of brucellosis in Hinggan League and provide insights into formulating prevention and control policies.

## Methods

### Case definition and data source

Case definition and data source were identical to those previously described [14]. The reported data on human brucellosis were extracted from the China Information System for Disease Control and Prevention, including the number of brucellosis cases grouped by sex, age, and occupation. The incidence rate was analyzed using software Excel 2016 (Microsoft, Redmond, WA, USA), and the epidemic characteristics were described using case number, constituent ratio, and rate. The National Health Commission of the People’s Republic of China determined that the collection of data from human cases of brucellosis was part of continuing public health surveillance of a notifiable infectious disease and was exempt from institutional review board assessment. The human brucellosis cases included in this study represent all regions in the Hinggan League (banners, counties, and cities). All data were supplied and analyzed in an anonymous format, without access to personal identifying information. Throughout the process, Microsoft Excel was used for data cleaning. Data were analyzed using SPSS17.0 (Chicago, IL, USA). *P* values < 0.05 were considered statistically significant.

### Bacterial strain identification

Blood samples were collected from 418 patients. Of these, 96% presented with low fever, sweat, fatigue, headache, and body pain, while more than 95% had been in close contact with animals (sheep). A total of 54 strains were examined, and these strains were obtained from six counties of Hinggan League from 2018 to 2019. All strains were recovered from 53 patients, of which two (XAMBs008 and XAMBs016) were obtained from the same patient. No animal strains were included in this study. The *Brucella* strains were isolated, and biotypes were identified using standard procedures [15, 16]. *Brucella melitensis* 16 M (BM), *B. abortus* 544 (BA), and *B. suis* 1330 (BS) reference strains were used as control strains. VITEK 2.0 Automated Bacteria Identification System (Bio-mérieux Inc., Durham, NC, USA.) was also used to assess these strains. Species-level identification was undertaken using *B. abortus*, *B. melitensis*, *B. ovis*, and *B. suis* PCR (AMOS-PCR) [17]. DNA was extracted with the Nucleic Acid Automatic Extraction System (LLXBIO China Ltd., Beijing, China) using a single loop of fresh bacterial cells that were grown for 48 h on *Brucella* agar (BD Difco/BBL). DNA concentrations were measured by UV spectrophotometry (NanoDrop 2000, Thermo Fisher Scientific, Waltham, MA, USA).

### MLVA genotyping of *Brucella* strains

A total of 54 strains were genotyped in this study; 49 of these strains were from five counties (banners/cities), but the origins of the remaining five strains were unknown. No other reference strains were included. MLVA was performed as previously described [18, 19]. The 16 primer pairs were divided into three groups: panel 1 (MLVA-8: eight loci including bruce06, bruce08, bruce11, bruce12, bruce42, bruce43, bruce45, and bruce55), panel 2A (three loci including bruce18, bruce19, and bruce21), and panel 2B (five loci including bruce04, bruce07, bruce09, bruce16, and bruce30); MLVA-11 (panels 1 and 2A), and MLVA-16 (panels 1, 2A, and 2B). PCR amplifications were performed in 20 μl reaction volumes. PCR products (5 μl) for the 16 loci were denatured and resolved by capillary electrophoresis on the ABI Prism 3130 Automated Fluorescent Capillary DNA Sequencer (Applied Biosystems, Foster City, CA, USA). Fragments were sized following comparison with a ROX (carboxy-X-rhodamine)-labeled molecular ladder (MapMaker 1000; Bioventures Inc., Murfreesboro, TN, USA) and Gene Mapper software version 4.0 (Applied Biosystems). The fragment sizes were subsequently converted to repeat unit numbers using a published allele numbering system [19, 20]. BioNumerics version 5.1 software (Applied Maths, Sint-Martens-Latem, Belgium) was used to analyze the MLVA-16 assay data (Table S1). Both categorical coefficient and unweighted pair group methods were applied to clustering analysis. Resultant genotypes were compared using the web-based Microbes Genotyping 2016–2018·V1.4.0 MLVA database (http://microbesgenotyping.i2bc.paris-saclay.fr/ databases/view/1156).

### Antimicrobial susceptibility testing

In vitro antimicrobial susceptibility testing of 25 randomly selected strains was performed as previously described [13, 21]*.* The minimum inhibitory concentrations (MICs [MIC_50_ and MIC_90_]) of doxycycline, tetracycline, gentamicin, ciprofloxacin, ofloxacin, moxifloxacin, streptomycin, rifampin, cotrimoxazole, and azithromycin against 25 *B. melitensis* were determined using the microdilution method (96-hole plate; Wenzhou Kont Biology and Technology Co., Ltd., Zhejiang, China) according to the Clinical and Laboratory Standards Institute (CLSI) guidelines [22]. The minimal inhibitory concentrations of cotrimoxazole is based on the trimethoprim (TMP) concentration in 1:19 combination with sulfamethoxazole (SMZ). The plates were incubated in ambient air at 35 °C and evaluated after 48 h. As the MIC breakpoints for clinically used antimicrobials have not yet been established for *Brucella*, the guidelines for a slow-growing bacterium (*Haemophilus influenzae*) were used as an alternative [22, 23]. The reference strains, *B. melitensis* 16 M and *H. influenza* ATCC 10211I*,* were used as quality control strains. The *Brucella* isolates were identified as resistant or susceptible to the antimicrobials based on the MIC, MIC_50_ and MIC_90_. These identifications were performed following the CLSI [22] using the criteria for slow-growing bacteria.

## Results

### Epidemic profile of human brucellosis during 2004–2019

**Fig. 1:**
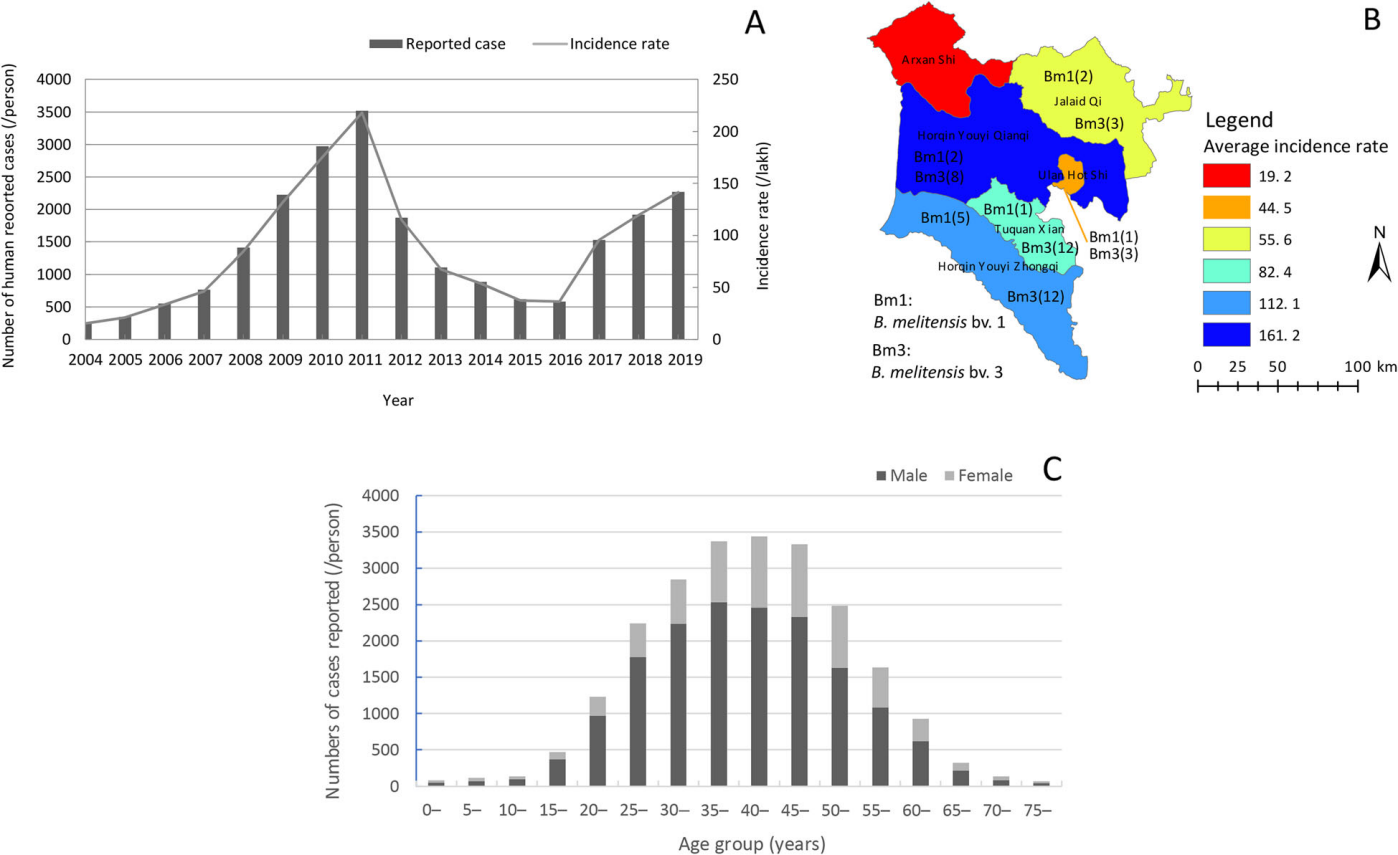
Demographic data of human brucellosis in Hinggan League during 2004–2019. **a** Annual reported cases and incidence rate (/100 000 people) of human brucellosis in Hinggan League during 2004–2019. **b** Average incidence rate of human brucellosis in various regions of Hinggan League during 2004–2019. **c** Distribution of human brucellosis cases at different ages and genders in Hinggan League during 2004–2019

A total of 22 848 cases of human brucellosis were reported from 2004 to 2019, with an incidence rate ranging from 15.4/100 000 to 141.4/100 000 during this period, and an annual average incidence of 87.1/100 000 in Hinggan League. Generally, the incidence of human brucellosis in Hinggan League significantly increased, from 15.4/100000 in 2004 to 218.1/100 000 in 2011, declining from 114. 8/100 000 in 2012 to 36.7/100 000 in 2016, and increasing again from 95.5/100 000 in 2017 to 141.4/100 000 in 2019. The largest number (3519) of cases occurred in 2011, with an incidence rate of 218.1/100 000, which was 14-fold the number reported in 2004 (15.4/100 000; Fig. 1a). The difference in incidence among the six regions was significant (*χ*^2^ = 32.561, *P* < 0.001).

### Distribution by region and time

From 2004 to 2019, the highest annual average incidence rate of human brucellosis (161.2/10 000) was in Horqin Youyi Qianqi. The annual average incidence rate in the remaining regions, from highest to lowest, was 112.1/100 000 in Horqin Youyi Zhongqi, 82.4/100 000 in Tuquan Xian, 55.6/100 000 in Jalaid Qi, 44.5/100 000 in Ulanhot Shi, and 19.2/100 000 in Arxan Shi (Fig. 1b). The incidence trends of this disease in six regions (counties, cities) in the Hinggan League were similar from 2004 to 2019, with the highest incidence rate observed between 2009 and 2011. Subsequently, although the number of cases in these regions had a decreasing trend from 2012 to 2016, the overall number of cases in each region was increased compared with 2004, especially, dramatically increasing again from 2017 to 2019 (Fig. S1). In addition, reported cases were observed every month, but the number of cases reported was significantly increased from March, and the reported case number was highest from May to August, accounting for 72.6% (16582/22 848) of cases. The incidence was high in summer and autumn, peaked in June, and gradually decreased in September (Fig. S2).

### Distribution of age, gender, and occupation

From the perspective of age of onset, human brucellosis in this study was found in all age groups (5–75 years) but was mainly concentrated in the 20–55 years old; the highest incidence was in the 40+ age group, accounting for 45.1% (10137/22 484) of all cases. The morbidity of males was higher than that of females in all age groups, and the differences in gender among the different age groups were statistically significant (*χ*^2^= 299.97, *P*< 0.001), with a male to female ratio of 2.69:1 (Fig. 1c). In terms of occupation, more than 18 occupations were noted in this population; the largest number of human brucellosis cases occurred in farmers, accounting for 89.8% (20520/22 848) of cases, followed by 902 cases in herdsmen, accounting for 4.0% (902/22 848) of cases. Disease occurrence in other occupations was fewer, accounting for ≤ 1.3% (285/22 848) of the cases (Table S2).

### Strain identification and genotyping characteristics of *B. melitensis*

A total of 54 *Brucella* strains were isolated and identified based on conventional biotyping methods, of which 13 were *B. melitensis* bv. 1, and the remaining 41 strains were *B. melitensis* bv. 3 (Table 1). All strains were positive for ProA, GlyA, TyrA, URE, and ELLM (Table S3). Based on AMOS-PCR, a 731 bp band was observed in 54 *Brucella* strains. Geographic distributions of the 54 strains are shown in Fig. 1b and Table S4.

**Table 1:** Biotyping characteristics of *Brucella* species isolates in this study

Code of isolate	No.	Growth characteristic				Monospecific sera			Phages lysis testing			Interpreted
		CO_2_ requested	H_2_S	BF	TH	A	M	R	Tb	BK_2_	Wb	
BA	1	+	+	+	–	+	–	–	CL	CL	CL	*B. abortus* 544
BM	1	–	–	+	+	–	+	–	NL	CL	NL	*B. melitensis*16M
BS	1	–	++	–	+	+	–	–	NL	CL	CL	*B. suis* 1330
Strains tested	13	–	–	+	+	–	+	–	NL	CL	NL	*B. melitensis* bv. *1*
	41	–	–	+	+	+	+	–	NL	CL	NL	*B. melitensis* bv. *3*

Description of data:

No., the number of strains tested

*BF* Basic fuchsin at 20 μg/ml (1/50 000, w/v), *TH* Thionin at 20 μg/ml (1/50 000, w/v)

Phages, *Tb* Tbilisi, *BK*_*2*_ Berkeley type 2, *Wb* Weybridge;

*CL* Confluent Lysis, *NL* No lysis;

+, positive (serum agglutination positive)

-, negative (serum agglutination negative)

**Fig. 2:**
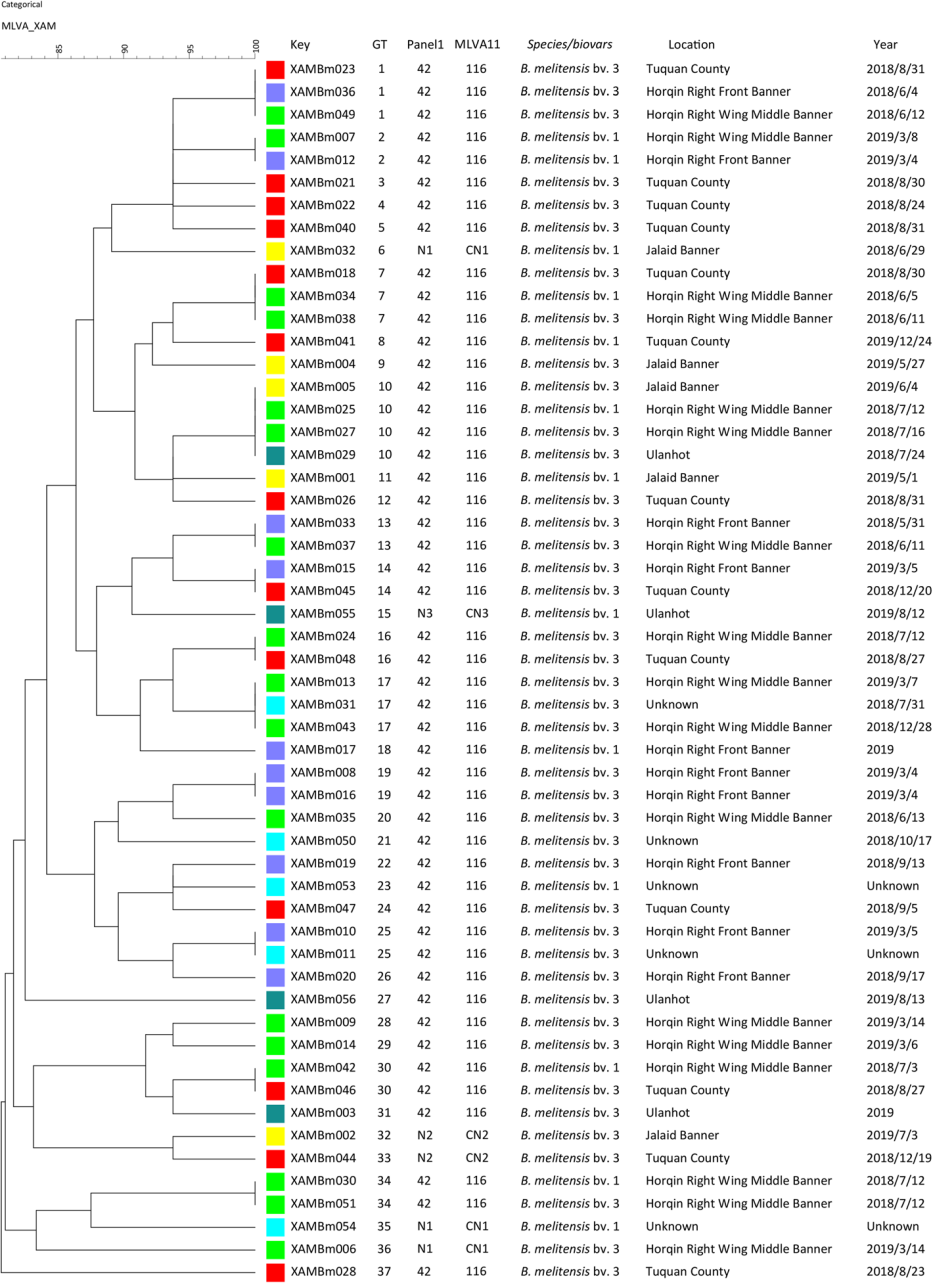
Dendrogram based on the MLVA-16 genotyping assay (UPGMA method), showing relationships between the 54 *B. melitensis* isolates. The columns show the identification numbers, MLVA-16 genotypes (GT), panel 1 genotypes and MLVA-11 (panels 1 and 2A) genotypes, species-biovar, their geographic location, and the year of isolation of the strains

Based on the eight VNTR loci, strains were divided into four panel 1 genotypes including 42 and N1–N3; 89% (48/54) of strains were panel 1 genotype 42 (Fig. 2 and Table S1). Similarly, these strains were clustered into four MLVA-11 genotypes (116 and CN1–CN3); 89% (48/54) of strains were MLVA-11 genotype 116, and the remaining three new genotypes were CN1–CN3 (Fig. 2 and Table S1). Based on the 16 VNTR loci, strains were grouped into 37 genotypes (GT1–37) with 80–100% genetic similarity, of which 12 were shared genotypes and 25 were single genotypes. A total of 10 (GT1, 2, 7,10, 13, 14, 16, 17, 25, and 30) of the 12 shared genotypes corresponded to 25 strains from two to three different regions that were isolated at similar times (Fig. 2).

### Antimicrobial susceptibility pattern of the 25 *B. melitensis* strains

The MIC range, and MIC_50_ and MIC_90_ values of the 25 *B. melitensis* strains are shown in Table 2. All 25 tested isolates were susceptible to doxycycline (MIC_90_, 0.1 μg/ml) and tetracycline (MIC_90_, 0.5 μg/ml), gentamicin (MIC_90_, 1 μg/ml), ciprofloxacin (MIC_90_, 1 μg/ml), ofloxacin (MIC_90_, 1 μg/ml), moxifloxacin (MIC_90_, 1 μg/ml), and streptomycin (MIC_90_, 2 μg/ml). In addition, 84% (21/25) of the strains were susceptible to rifampin, and 16% (4/25) of the strains exhibited intermediate susceptibility. The MIC values of cotrimoxazole ranged from 1/19 to 4/76 μg/ml (MIC_90_, 4/76 μg/ml), with 20% (5/25) of the strains exhibiting resistance to cotrimoxazole. All 25 strains were resistant to azithromycin (MIC_90_, 64 μg/ml). No association between biovar type and the susceptibility profile of the tested *B. melitensis* strains was observed.

**Table 2:** MIC range, MIC_50_, and MIC_90_ of 10 antimicrobial agents against 25 *B. melitensis* strains

Antibiotic	MIC (μg/ml)			Classification of isolates, ^1^No. (%)			Breakpoints (μg/ml)		
	Range	50%^†^	90%^†^	S	I	R	S	I	R
Doxycycline	0.12–0.24	0.12	0.12	25 (100)	0	0	≤ 1	–	–
Tetracycline	0.25–1	0.25	0.5	25 (100)	0	0	≤ 1	–	–
Gentamicin	0.5–1	0.5	1.0	25 (100)	0	0	≤ 4	–	–
Ciprofloxacin	0.5–1	0.5	1.0	25 (100)	0	0	≤ 1	–	–
Ofloxacin	1.0	1.0	1.0	25 (100)	0	0	≤ 2	–	–
Moxifloxacin	1.0	1.0	1.0	25 (100)	0	0	≤ 1	–	–
Streptomycin	2–4	2.0	4.0	25 (100)	0	0	≤ 8	–	–
Rifampin	0.08–3.2	0.5	1.0	21 (84)	4 (16)	0	≤ 1	2	≥ 4
Cotrimoxazole	1/19–4/76	2/38	4/76	20 (80)	0	5 (20)	≤ 2/38	–	–
Azithromycin	16–64	64	64	0	0	25 (100)	≤ 4	–	–

Note: *S* susceptible, *I* intermediate susceptibility, *R* resistant, ^*1*^*No*. number of isolates

^†^50 and 90%, MIC at which 50 and 90% of the isolates are inhibited. −: Not determined

Cotrimoxazole: Trimethoprim/sulfamethoxazole (only the trimethoprim portion of the 1/19 drug ratio is displayed)

Breakpoints for slow-growing bacteria according to CLSI recorded for *Haemophilus* spp. were provided

## Discussion

Hinggan League is one of the most important animal husbandry production bases in the Inner Mongolia Autonomous Region of China, a historically endemic area of animal and human brucellosis. Animal and human brucellosis is still circulating throughout the region. Therefore, the spread of brucellosis will result in heavy economic losses to the livestock industry and poses a serious health threat to the human population. In this study, the epidemiology of human brucellosis and in vitro antimicrobial susceptibility characteristics of *B. melitensis* isolated from this region were determined. The results showed that the incidence rate of human brucellosis in the Hinggan League from 2004 to 2019 exhibited a significant increasing trend and imbalance in distribution among regions. The increasing trend in incidence rate of human brucellosis in this region coincides with previous studies [24], the rate of human brucellosis infection has rapidly increased in recent years. Tongliao City is one of the highest risk areas for human brucellosis in Inner Mongolia (China), as Tongliao city is geographically close to Hinggan League, incidence rates increased dramatically from 9.2/100 000 in 2007 to 69.2/100 000 in 2011, then decreased from 2012 to 2016; incidence rates rose again in 2017 to 44.3/100 000 [25]. The incidence of human brucellosis was high from 2014 to 2018 in Huludao, China, primarily due to contact with domestic animals without sufficient protective measures [26]. Previous investigation results [27] showed that serum samples from 226 human, 669 sheep, and 54 calves were collected from 12 villages in Horqin Youyi Zhongqi and Tuquan Xian in 1987, and no positive samples were detected except one sample from sheep from Horqin Youyi Zhongqi. In 1988, a total of 821 serum samples (717 in sheep, 21 in calves, and 83 in humans) from Ulanhot Shi were collected and tested, and no positive samples were detected. In this study, the highest incidence of human brucellosis was in Horqin Youyi Qianqi and Horqin Youyi Zhongqi and the lowest incidence rate in Arxan Shi. The highest incidence rate of human brucellosis was observed among the two above regions, which are traditionally pastoral areas, and animal husbandry is great developed. Breeding and animal production are the main source of revenue for local persons, but Arxan Shi is a forest region with less animal breeding than other regions.

All ages are susceptible to brucellosis, with most cases occurring in young and middle-aged people. In addition, there are more cases of males than females in different age groups, which may be related to the fact that men are more involved in livestock production and have more exposure to the source of infection. Humans brucellosis without gender and age differences, infected mainly depending upon the exposure opportunities. Farmers and herdsmen comprise the majority of infected cases, as they are regularly in contact with infected animals. This result was consistent with data from Tongliao City, Inner Mongolia [25], most of these were agriculturalists (81.9%) and pastoralists (12.4%), aged 25–59 years (85.4%); the male-to-female ratio was 2.64:1. The proportion of students is second only to that of farmers and herdsmen, Mongolian boys have the custom of playing with lambs, which may increase their risk of infection. Moreover, some brucellosis cases were reported in administrative cadres and teachers, who was non-occupational population for brucellosis; this suggests that brucellosis in this region has an expansive trend from farmers and herdsmen to non-occupational, which may be related to changes in livestock infected transfer and dietary structure [28]. Similarly, a study from Iran showed that most patients lived in rural areas, and that 20.8% (17.4–24.2%) of the patients were ranchers and farmers, 16.9% (14.5–19.4%) were students, and 31.6% (27.0–36.2%) were housewives [29].

In this study, all *Brucella* isolated from patients were identified as *B. melitensis* (bv. 1 and bv. 3), strains were extensively distributed in five areas (total of six areas), and *B. melitensis* bv. 3 was the dominant species in the area examined. These results coincided with the serious human brucellosis epidemic [30], for which *B. melitensis* bv. 3 caused the majority of human brucellosis cases in northern and southern China [31–33]. However, 89% (48/54) of strains in this study carried MLVA-11 genotype 116; strains with this genotype have a vital epidemiological significance to the humans infected brucellosis [34]. A total of 54 strains were grouped into 12 shared genotypes, these genotypes represented 29 strains, and the genetic similarity coefficient among the strains was higher than 80%. This suggested that the human brucellosis outbreak occurred from strains from a common ancestor. Moreover, 10 shared genotypes comprised strains from two to three different regions with similar isolation times, indicating that the human brucellosis outbreak epidemic resulted from infected animal cross-transfer among different regions. This result was consistent with a previous study, which MLVA-genotyped 116 human *B. melitensis* strains in Ulanqab, Inner Mongolia, and demonstrated that a multipoint-outbreak epidemic originated from multiple common sources [35]. A strict control strategy in infected animals (sheep) transfer and trade in this region is urgent.

In the present study, doxycycline, which is the most commonly used therapeutic agent against *Brucella* infection, had the lowest MIC_50_ and MIC_90_ values; the MIC_50_ and MIC_90_ of doxycycline were identical. A previous study reported [36] that doxycycline had the lowest MIC_50_, while rifampicin had the highest; four strains were non-susceptible to rifampicin; and one strain was resistant to trimethoprim-sulphamethoxazole. In another study, 355 *Brucella* strains were susceptible to trimethoprim-sulfamethoxazole, tetracycline, doxycycline, streptomycin, and ciprofloxacin, while 277 (64%) isolates were probably resistant to rifampin, and 7 (2%) isolates were probably resistant to ceftriaxone [37]. In this study, 16% (4/25) of the strains exhibited intermediate susceptibility to rifampin (2–3.2 μg/ml). Similarly, *B. melitensis* resistance to rifampicin was observed in Kazakhstan, where more than 50% isolates were rifampicin-resistant [38]. Moreover, *Brucella* isolates had decreased sensitivity to rifampin was found in 35.1% of the isolates in Iran [39]. In this study, azithromycin had the highest MIC_50_ and MIC_90_ values. A related reported from Liaoning, China, showed that 31 *B. melitensis* isolates were complete resistant to azithromycin, and a T/C SNP alteration was identified at position 2632 of the 23S RNA gene in strains resistant to azithromycin [12]. It was shown that, although a short oral course of azithromycin significantly reduced infection severity, this did not treat animals as effectively as the classic regimen of doxycycline over a longer period of time [40]. However, Landínez R et al. [41] reported MIC_90_ values of 0.5–1.00 for azithromycin in 358 pathogenic *B. melitensis* strains isolated from three different Spanish regions; the sensitivities of these strains to azithromycin and tetracycline were similar. Denk Affan et al. [42] suggested a combination of quinolones and azithromycin as an alternative to doxycycline and rifampicin for the treatment of brucellosis. These results indicate that further investigations of the possible therapeutic roles of azithromycin for human brucellosis are warranted.

Moreover, Hinggan League is a developed area of animal husbandry, and the large number of antibiotics used in the breeding industry may increase the risk that *Brucella* strains become less sensitive to various antibiotics. As increasing resistance to rifampin and cotrimoxazole has been reported in many parts of the world, we suggest periodic reassessments of strain susceptibility to those antibiotics used most frequently in treatment. This will support the early detection of any drug resistance, especially in areas of endemicity.

Our study had several limitations. First, the epidemiological data were collected through passive public health surveillance. Thus, the data may be influenced by underreporting, the attending physician’s misunderstanding of the disease, and laboratory misdiagnoses. Second, the susceptibility of 25 *B. melitensis* strains to 10 antibiotic agents was tested in vitro. Although a variety of these antimicrobial agents appeared to be active, further investigation of antibiotic susceptibility in more strains is needed. Finally, no animal strains were isolated in this study. Investigations of genetically related strains between human and animals might clarify brucellosis epidemiology.

## Conclusions

Generally, the incidence rate of human brucellosis in the region examined exhibited an increasing trend from 2004 to 2019, although it decreased between 2012 and 2016. *B. melitensis*, particularly biovar 3, was the etiological agent most frequently isolated from humans in this region. This suggested that sheep were the principal cause of human brucellosis. Moreover, MLVA genotyping indicated that the human brucellosis outbreak was due a single source of infection. These data suggested that the launch of comprehensive strategies for prevention and control in this region, especially limiting the movement of infected sheep, is urgent.

## Supplementary information

**Additional file 1: Table S1.** Table representing strain identification codes (Key), GT (MLVA-16), MLVA-8, MLVA-11, biovars, host, regions, and year of isolation for 54 *B. melitensis* isolates.

**Additional file 2: Table S2.** Occupation distribution of human brucellosis in this study. **Table S3.** Biochemical characteristics of 54 *Brucella* strains identified by VETEK 2.0. **Table S4.** Location, species/biovars, and numbers of *B. melitensis* in this study

**Additional file 3: Figure S1.** Incidence rates of human brucellosis in various regions of Hinggan League during 2004–2019.

**Additional file 4: Figure S2.** Time distribution of human brucellosis cases at different months in Hinggan League during 2004–2019.

## Data Availability

The data sets supporting the results of this article are included within the article and its additional files.

## Abbreviations

AMOS-PCR: AMOS (*abortus*, *melitensis*, *ovis,* and *suis*)-polymerase chain reaction
MLVA: Multiple locus variable-number tandem repeat analysis
WGS: Whole genome sequencing
SNP: Single nucleotide polymorphism
MIC: Minimum inhibitory concentration
CLSI: The Clinical and Laboratory Standards institute
TMP: Trimethoprim
SMZ: Sulfamethoxazole
