# Supplementary material for: Epidemiologically characteristics of human brucellosis and antimicrobial susceptibility pattern of Brucella melitensis in Hinggan League of the Inner Mongolia Autonomous Region, China
Source: Infect Dis Poverty. 2020 Jun 29;9:79. doi: 10.1186/s40249-020-00697-0 (PMC7325291; doi:10.1186/s40249-020-00697-0)
Supplement: Supplementary file 2 — Additional file 2: Table S2. Occupation distribution of human brucellosis in this study. Table S3. Biochemical characteristics of 54 Brucella strains identified by VETEK 2.0. Table S4. Location, species/biovars, and numbers of B. melitensis in this study [file 40249_2020_697_MOESM2_ESM.docx]

Supplementary Table 2. Occupation distribution of human brucellosis in this study

| Occupation | Number of cases | Percentage (%) |
| --- | --- | --- |
| Waiter in public places | 0 | 0.00 |
| Daycare worker | 1 | 0.00 |
| Fisherman | 1 | 0.00 |
| Sailor and long-distance driver | 1 | 0.00 |
| Catering staff | 9 | 0.04 |
| Medical staff | 12 | 0.05 |
| Teacher | 19 | 0.08 |
| The emeritus and retired | 30 | 0.13 |
| Business person | 33 | 0.14 |
| Civil servants and staff | 35 | 0.15 |
| Childcare children | 39 | 0.17 |
| Rural laborer | 62 | 0.27 |
| Unknown | 93 | 0.41 |
| Scattered children | 106 | 0.46 |
| Others | 167 | 0.73 |
| Worker | 231 | 1.01 |
| Student | 285 | 1.25 |
| Housekeeping, housework  and unemployment people | 302 | 1.32 |
| Herdsman | 902 | 3.95 |
| Farmer | 20520 | 89.81 |
| Total | 22848 | 100.00 |

Supplementary Table 3. Biochemical characteristics of 54 *Brucella* strains identified by VETEK 2.0

| Key | Strains No. | ProA | GlyA | TyrA | URE | ELLM | APPA | AGLU | ILATk | SUCT |
| --- | --- | --- | --- | --- | --- | --- | --- | --- | --- | --- |
| 16M | 1 | + | + | + | + | + | - | - | - | - |
| 544 | 1 | + | + | + | + | - | - | - | + | - |
| 1330 | 1 | + | + | + | + | - | + | - | - | - |
| Tested  strains | 43 | + | + | + | + | + | - | - | - | - |
|  | 1 | + | + | + | + | + | - | + | - | - |
|  | 1 | + | + | + | + | + | + | - | - | - |
|  | 5 | + | + | + | + | + | + | + | - | - |
|  | 1 | + | + | + | + | + | + | + | + | - |
|  | 2 | + | + | + | + | + | + | - | + | - |
|  | 1 | + | + | + | + | + | - | - | - | + |
| Total | 54 | 54 | 54 | 54 | 54 | 54 | 10 | 7 | 3 | 1 |

Note: +, positive reaction; -, negative reaction.

Supplementary Table 4. Location, species/biovars, and numbers of *B. melitensis* in this study

| Location | Species-biovars | Number | Total |
| --- | --- | --- | --- |
| Horqin Youyi Qianqi | *B. melitensis* bv.1 | 2 | 10 |
|  | *B. melitensis* bv.3 | 8 |  |
| Horqin Youyi Zhongqi | *B. melitensis* bv.1 | 5 | 17 |
|  | *B. melitensis* bv.3 | 12 |  |
| Jalaid Qi | *B. melitensis* bv.1 | 2 | 5 |
|  | *B. melitensis* bv.3 | 3 |  |
| Tuquan Xian | *B. melitensis* bv.1 | 1 | 13 |
|  | *B. melitensis* bv.3 | 12 |  |
| Ulanhot Shi | *B. melitensis* bv.1 | 1 | 4 |
|  | *B. melitensis* bv.3 | 3 |  |
| Unknown | *B. melitensis* bv.1 | 2 | 5 |
|  | *B. melitensis* bv.3 | 3 |  |
