# Supplementary figures and images for: Epidemiologically characteristics of human brucellosis and antimicrobial susceptibility pattern of Brucella melitensis in Hinggan League of the Inner Mongolia Autonomous Region, China
Source: Infect Dis Poverty. 2020 Jun 29;9:79. doi: 10.1186/s40249-020-00697-0 (PMC7325291; doi:10.1186/s40249-020-00697-0)

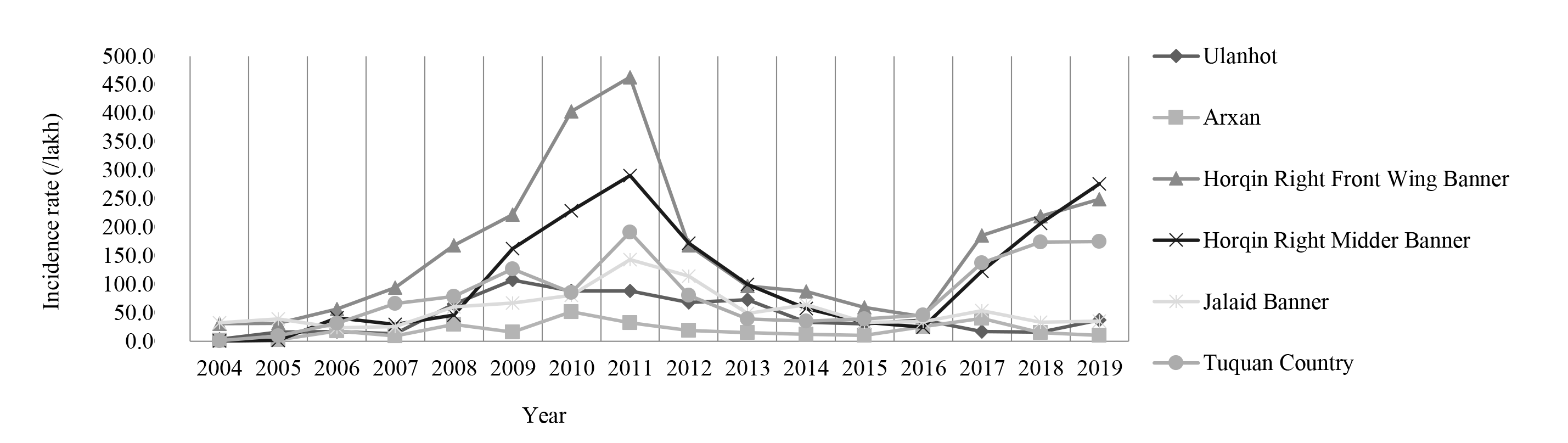

Supplement: Supplementary file 3 — Additional file 3: Figure S1. Incidence rates of human brucellosis in various regions of Hinggan League during 2004–2019. [file 40249_2020_697_MOESM3_ESM.tif]

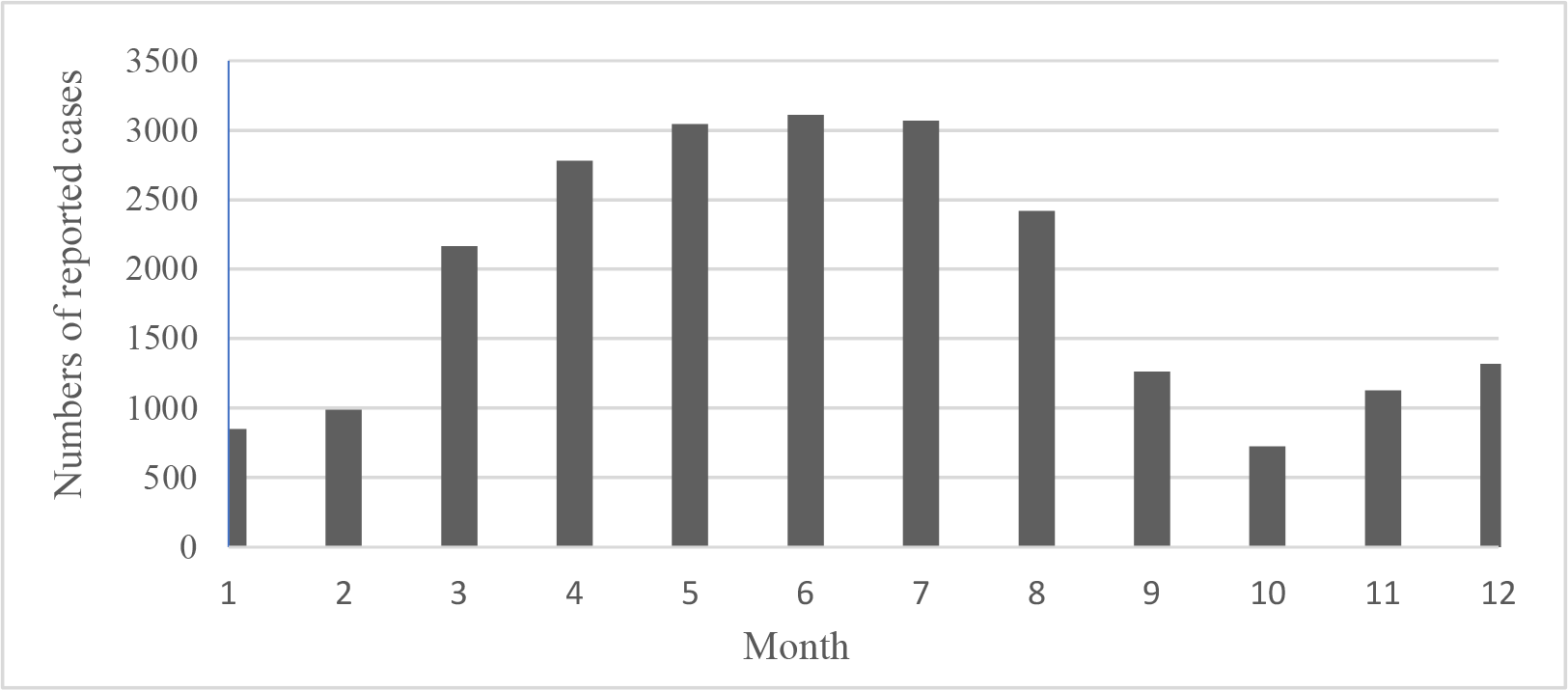

Supplement: Supplementary file 4 — Additional file 4: Figure S2. Time distribution of human brucellosis cases at different months in Hinggan League during 2004–2019. [file 40249_2020_697_MOESM4_ESM.tif]
